# Barriers and facilitators to parent-delivered interventions for children with or infants at risk of cerebral palsy. An integrative review informed by behaviour change theory

**DOI:** 10.1080/09638288.2024.2338193

**Published:** 2024-04-16

**Authors:** Jill Massey, Phillip Harniess, Deborah Chinn, Glenn Robert

**Affiliations:** a Evelina London Children’s Hospital, Guys and St Thomas’ NHS Foundation Trust, London; b Florence Nightingale School of Nursing Midwifery and Palliative Care, James Clerk Maxwell Building, London; c Great Ormond Street Hospital, London; d University of Exeter, Peninsula Childhood Disability Research Unit (PenCRU), Medical School, Exeter, Devon, UK

**Keywords:** Parents, child, cerebral palsy, intervention, participation, rehabilitation, parent-delivered, therapy

## Abstract

**Purpose:**

Empowering parents to deliver evidenced-based interventions improves outcomes for children with or infants at risk of cerebral palsy (CP), by integrating repetition and contextual learning into daily routines. We aimed to identify the barriers and facilitators to parent-delivered interventions and suggest practice improvements guided by behaviour change models.

**Methods:**

Eight electronic databases were searched to identify studies presenting parent and therapist perspectives on parent-delivered interventions in CP. Included studies were critically appraised using validated checklists. Barriers and facilitators to parent-delivered interventions were identified and categorised into subcomponents of The Capability Opportunity and Motivation Model of Behaviour (COM-B), the Theoretical Domains Framework (TDF) and the Behaviour Change Wheel to formulate appropriate practice recommendations.

**Results:**

Thirty-four studies were identified which mainly used qualitative or randomised control trial designs. Barriers to parent-delivery included insufficient parental knowledge, lack of confidence and time. Facilitators included staff continuity, empowering parents, efficient resource utilisation and flexible delivery. Practice recommendations emphasise realistic goal setting, tailored parental education and enhancing the coaching skills of therapists.

**Conclusions:**

Fostering parent-delivered interventions requires addressing knowledge gaps, skill and capacity of parents and therapists. Therapists forming strong alliances with parents and setting collaborative realistic goals are key to successful parent-delivered interventions.

## IMPLICATIONS FOR REHABILITATION

- Enhancing parents’ skills and knowledge regarding CP interventions can increase their confidence (psychological capability) in actively participating in intervention delivery.
- Structuring services to accommodate family schedules and providing adequate resources (physical and social opportunity) reduce the burden on families and facilitate their engagement.
- Providing training and resources to therapists enhances their skills and knowledge (psychological capability) in coaching and educating parents effectively.
- Emphasizing the value of collaborative goal setting in achieving positive outcomes for the child and acknowledging progress (reflective and automatic motivation) motivates both parents and therapists to actively engage in intervention delivery.

## Introduction

Cerebral palsy (CP) is the most common physical disability in childhood [1,2]. The prevalence of CP is estimated to be between 1.4 cases per 1000 live births in high income countries [1]. CP is an overarching term for a heterogeneous group of “permanent but not unchanging disorders of movement and posture caused by damage to the developing brain” [3]. The impact of CP can be seen across a person’s lifespan and influences independence and participation in education, social and community activities [4]. Most often, movement difficulties are identified, but children with CP may also experience challenges with pain, communication, vision, hearing, behaviour and sleep [5].

Systematic reviews and international clinical practice guidelines identify several effective activity-based interventions for children with and infants at high risk of CP [6, 7]. Key features of successful interventions include a focus by the clinician on family-set goals, intensive practice in a real-life context, and a team approach [6, 7]. Robust evidence shows repetition and task practice increase the likelihood of positive outcomes from these programmes [8, 9]. Parent-delivery has been identified as a key component to all interventions for children with CP [6]. This review focusses on parent-delivered interventions. They are defined here as interventions that have been taught or shown to the parent to enable them to deliver them regularly at home and/or within the community [10].

Given the scarcity of statutory resources and availability of health care professionals (HCPs), it is challenging to achieve the intervention dosages and repeated practice required within the child’s day. One approach to maximise the intensity and contextual practice opportunities for children is to support parents (or caregivers) to deliver these interventions at home. The term ‘parent’ will be used throughout this review for simplicity to include any main caregivers who have a similar parental role.

Parent-delivered interventions have been shown to be at least as effective as therapist delivered interventions [10], whilst achieving greater levels of intensity and therefore more positive outcomes for children [11–13]. Internationally, whilst the importance of involving family to promote shared decision-making with healthcare professionals has been recognised, it appears to remain limited in clinical practice [11, 14, 15]. Equally, whilst HCPs recognise the expert knowledge that parents bring in terms of their child’s needs and capabilities, [15] they often report a lack of confidence in their own abilities to adequately equip families to deliver interventions [16].

Milton and Roe [17] reviewed quantitative studies of parent-delivered, best-evidenced intervention approaches for children with hemiparesis, including bimanual therapy, Constraint Induced Movement Therapy (CIMT) or modified Constraint Induced Movement Therapy (mCIMT) home programmes. The review concluded that successful therapist behaviours involved working in partnership, employing collaborative goal setting, and matching goals to intervention activities to help parents grade them appropriately. A later meta-ethnographic review of the determinants of parent involvement in all types of parent-delivered therapy interventions for children with CP [18] analysed qualitative and mixed methods studies published up until 2017. This paper reported the importance of parents feeling empowered, motivated, and having a good therapeutic relationship. The authors also developed a reflective checklist based on review findings for therapists to use before initiating parent-delivered therapy [10].

Research suggests that to ensure the successful delivery of interventions by parents, it is likely that a shift in both parent and therapist behaviour is needed [19, 20]. While previous reviews [18], Milton [17], Harniess [21] have advanced our overall understanding of parent-delivered and parent engagement in rehabilitation, they did not systematically explore parent-delivered interventions through a lens of contemporary behaviour change theory. It is of value to both clinical practice and research to apply such theories to the field of parent-delivered interventions, as they can provide a better understanding of the relevant barriers and facilitators for stakeholders to achieving service improvements. Therefore, our review aimed to add to the literature by integrating contemporary qualitative and quantitative studies to identify the barriers and facilitators to parent-delivered interventions for children with or infants at high risk of CP. The Capability Opportunity Motivation-Behaviour (COM-B) and Theoretical Domains Framework (TDF) and the Behaviour Change Wheel (BCW) [22] were used to frame identified barriers and facilitators to parent-delivered interventions in behavioural terms. Developed by expert consensus from a range of psychological and organisational theories, the COM-B and TDF give insight into the determinants of people’s behaviour and suggest theoretical approaches to encourage behaviour change across a range of stakeholders. The BCW also provides guidance on specific strategies that can be used to modify target behaviours. The COM-B and TDF have been used previously in studies aiming to improve the health and safety of children and their families by identifying barriers and facilitators to certain behaviours [23–25].

## Review questions

- What are the barriers and facilitators to parent-delivered interventions for children with or infants at risk of cerebral palsy associated with the TDF and COM-B models of behaviour change?
- What practice recommendations can be made to enhance parent-delivered interventions utilising guidance from the Behaviour Change Wheel?

## Methods

To date, quantitative studies in this area have tended to focus on child outcomes and the feasibility of parent-delivered interventions rather than exploring the parental experiences and the wider impact of intervention delivery on families [21]. The integrative review method allows for combining a range of methodologies to integrate conceptual findings rather than to aggregate outcome data [26]. The review was guided by strategies proposed by Whittemore and Knafl (2005) [27].

**Figure 1.**
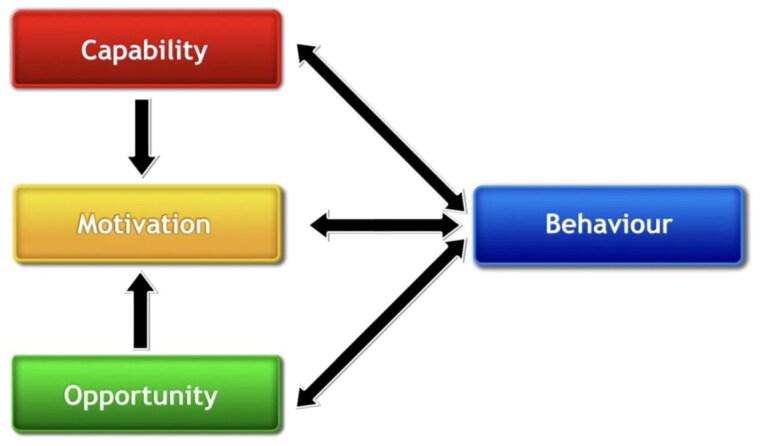
Capability opportunity motivation behaviour (COM-B).

The COM-B model [22] (Figure 1), was used as the framework for this review. It is a theory of behaviour that proposes three main components that influence behaviour.

**Figure 2.**
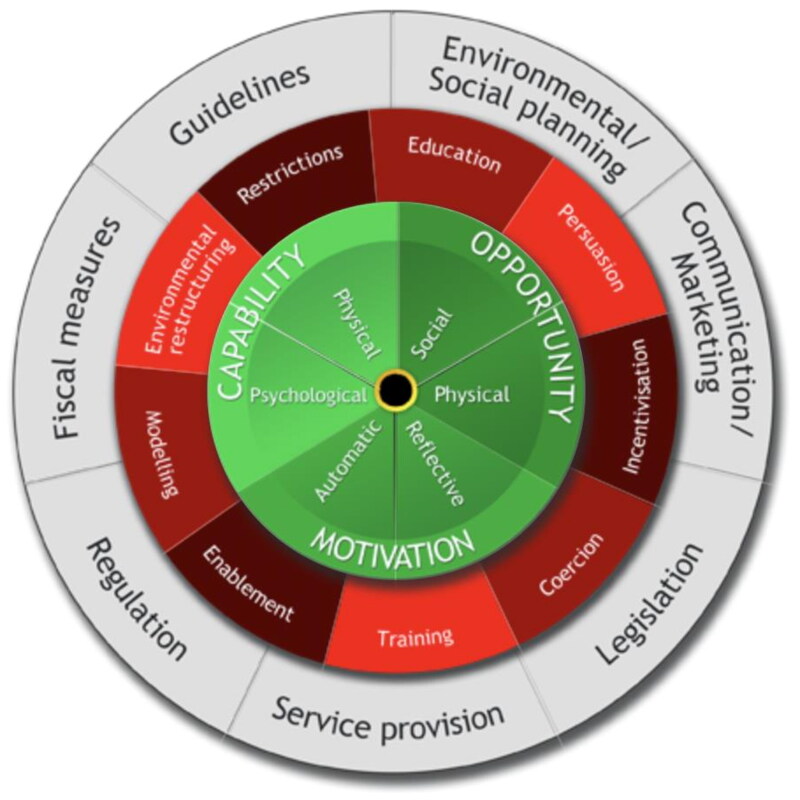
Behaviour change wheel [22].

These are: Capability (C), Opportunity (O) and Motivation (M). [22] Capability can either be psychological (e.g. knowledge or memory) or physical (e.g. skills or stamina); opportunity can be social (e.g. societal influences) or physical (e.g. environmental resources); and motivation can be automatic (e.g. emotion) or reflective (e.g. beliefs, intentions). Behaviour (B) occurs as the result of an interaction between these three components. The Theoretical Domains Framework (TDF) synthesises multiple theoretical constructs which explain behavioural change into 14 domains [28]. The TDF was used to highlight potential behavioural targets alongside COM-B (Table 1). The COM-B and TDF components were then mapped onto the Behaviour Change Wheel (BCW) to guide practice recommendations (Figure 2).

**Table 1.** COM-B model and TDF domains and definitions (Michie et al. 2014) [22].

COM-B component	TDF domains	Definition
Capability	Skills	An ability of or proficiency acquired through practice (skills, skills development, competence, ability, practice, skill assessment)
	Knowledge	An awareness of the existence of something (including knowledge of condition/scientific rationale, procedural knowledge, knowledge of task environment)
	Memory, attention, decision	The ability to retain information, focus selectively on aspects of the environment and choose between two or more alternatives (memory, attention, attention control, decision making, cognitive overload/tiredness)
	Behavioural regulation	Anything aimed at managing or changing objectively observed or measured actions (self-monitoring, breaking habit, action planning)
Opportunity	Social influences	Those interpersonal processes that can cause individuals to change their thoughts, feeling, or behaviours (social pressure, social norms, group conformity, social comparisons, groups norms, social support, power, intergroup conflict, alienation, group identity, modelling)
	Environment context and resources	Any circumstance of a person’s situation or environment that discourages or encourages the development of skills and abilities, independence, social competence, and adaptive behaviour (environmental stressors, resources/material resources, organisational culture/climate, salient events/critical incidents, person x environment interaction, barriers and facilitators)
Motivation	Beliefs about capabilities	Acceptance of the truth, reality, or validity about an ability, talent, or facility that a person can put to constructive use (self-confident, perceived competence, self-efficacy, perceived behavioural control, beliefs, self-esteem, empowerment, professional confidence)
	Beliefs about consequences	Acceptance of the truth, reality, or validity about outcomes of a behaviour in a given situation (beliefs, outcome expectancies, characteristics of outcome expectancies, anticipated regret, consequents)
	Social/professional identity	A coherent set of behaviours and displayed personal qualities of an individual in a social or work setting (professional identity, professional role, social identity, identity, professional boundaries, professional confidence, group identify, leadership, organisational commitment)
	Optimism	The confidence that things will happen for the best or that desired goals will be attained (optimism, pessimism, unrealistic optimism, identity)
	Intentions	A conscious decision to perform a behaviour or a resolve to act in a certain way (stability of intentions, stages of change model, trans theoretical model and stages of change)
	Goals	Mental representations of outcomes or end states that an individual wants to achieve (goals (distal/proximal), goal priority, goal/ target setting, goals (autonomous/controlled), action planning, implementation intention)
	Reinforcement	Increasing the probability of a response by arranging a dependent relationship, or contingency, between the response and a given stimulus (rewards (proximal/distal, valued/not values, probable/improbable), incentives, punishment, consequents, reinforcement, contingencies, sanctions)
	Emotion	A complex reaction pattern, involving experiential, behavioural, and physiological elements, by which the individual attempts to deal with a personally significant matter or event (fear, anxiety, affect, stress, depression, positive and negative affect, burn-out)

A recent methodological review of studies investigating barriers and facilitators in healthcare criticised the poor operationalisation of these terms [29]. For the purposes of this review, the term ‘barrier’ is defined as any factor that makes it difficult for parents to deliver their child’s interventions. This aligns with other definitions where barriers are defined as anything that makes it more difficult for individuals to access, use or benefit from healthcare [30]. A ‘facilitator’ was defined as a factor that either supports or makes parent involvement easier [31]. The review protocol was registered with Prospero 2021 CRD42021262573.

### Identification of the research problem

Research investigating the barriers and facilitators to implementing parent-delivered interventions for children with or infants at risk of cerebral palsy has not been synthesised. The integrative review aimed to address this gap in the literature by identifying these barriers and facilitators linked to the TDF and COM-B models of behaviour change. Additionally, the study sought to make practice recommendations to improve parent-delivered interventions utilising guidance from the Behaviour Change Wheel.

### Literature search

The search included variations of the following terms: cerebral palsy, motor disorders, parent or caregiver and rehabilitation. Terms were combined using Boolean operators ‘OR’ and ‘AND’ and were searched as keywords and Medical Subject Headings (MeSH). The search was originally created for MEDLINE and then adapted for PubMed, CINAHL, PsycINFO, Embase, Scopus, Ovid and Cochrane. The full search strategy for MEDLINE can be found in supplementary material (Appendix 1). The search included articles from 1991 to January 2023 and was not restricted by study type. This period was selected to allow for changes in intervention approaches and family centered practice to be considered. Reference lists of included studies and selected reviews were also hand searched for eligible studies. The review only included peer-reviewed articles and excluded grey literature including dissertations, conference abstracts, guidelines and policy documents. Systematic reviews that were identified were checked against the criteria for this review. Where not all included studies met the criteria, the reference lists were checked to allow the inclusion of individual studies. Studies that were not published in English language were also excluded.

### Study selection

The resulting studies were screened for the inclusion/exclusion criteria shown in Table 2.

**Table 2.** Inclusion and exclusion criteria.

	Inclusion criteria	Exclusion criteria
Population	Parents or primary caregivers of children (under 18 years) with or at risk* of CP and who had been involved in the delivery of their child’s rehabilitation.	Studies which did not include parents or caregivers of children with CP
Intervention	Intervention: parent-delivered rehabilitation** for their child(ren) where rehabilitation was led or defined by the therapist with parental involvement in any setting (home, virtual, clinic).	Intervention was delivered by school/teaching or therapy assistants or by the therapist only
Outcomes	Studies which had elicited views of either parent(s)/caregiver(s) and therapists on parents’ experience of participating in the delivery of CP therapy.	Papers which did not include either parent or therapist perspectives on parental participation in therapy

*At risk of CP defined as ‘neuroimaging indicating likely motor impairment or absence of fidgety movements as determined through the General Movements Assessment’ (Prechtl, 1997, 2001).

** Defined as interventions, which targeted the International Classification Function domains of body structures and functions, activity and participation.

A decision was made to also include studies whose populations were not exclusively parents of children with or infants at high risk of CP, but only if these populations comprised less than 20% of the overall sample [32, 33]. This decision was taken because it was felt that such studies would still contribute valuable data on parent-delivered interventions in CP. However, caution was employed when extrapolating the findings of mixed population samples when these were not presented separately. Included in the analysis were studies incorporating perspectives from therapists alongside those from parents [34–38] with therapist viewpoints also extracted for data synthesis.

### Screening

All articles resulting from the searches were imported and managed through the Covidence online platform. Duplicates were removed. First and second authors (JM, PH) independently screened titles and abstracts, with any disagreements resolved by discussion. Full text articles were then independently screened by first and second authors to gain final consensus on which articles to include.

#### Quality appraisal

Independent appraisal of the studies was conducted by the first author (JM) and a sample of seven papers (20%) was reviewed by the second author (PH) for rigour. The Critical Appraisal Skills Programme (CASP) checklists were used for qualitative [39] and cohort studies [40] and Randomised Control Trials (RCT) [41]. The ROBINS-I tool was used for non-randomised experimental studies [42]. Surveys were assessed using a Critical Appraisal (Survey) tool from the Centre for Evidence-Based Management (CEBMa) [43]. The tools were not used to provide an overall score for each study, but instead to assess the overall quality of the data retrieved to answer the review questions and in the small number of lower quality studies [44, 45] to carefully consider the validity of their findings.

#### Data extraction

A standardized data extraction form was devised by the first author (JM) to collect study characteristics, namely: author, year, country, aims, design, methods, setting, sample, intervention type and findings on outcome measures (qualitative and quantitative data). The first author and the second author were allocated 65% (*n* = 22) and 35% (*n* = 12) of the studies, respectively, to independently extract the study details. In addition, a subsample of 20% of the total studies (*n* = 7) which featured a range of methodologies (three qualitative and four quantitative) underwent data extraction by both reviewers, who then met to discuss and resolve discrepancies or areas of uncertainty.

#### Data analysis

The 34 studies were analysed through coding of themes developed in each qualitative aspect of the studies and the statistically significant results in the quantitative aspect of the studies. A constant comparison method guided the analysis, which “*converts extracted data into systematic categories, facilitating the distinction of patterns, themes, variations, and relationships.*” [27] This method involves iterative comparisons across data sources including data reduction, display, comparison, conclusions, and verification. Data reduction commenced through the extraction of relevant first-order constructs (parent and therapists’ words/feedback) and second order constructs (authors’ interpretation of those words/feedback) to answer the research question [27, 46]. Tabulation was used to display the data at an individual study level then displayed in a combined matrix for comparison. The lead researcher developed codes based on the research questions. New codes were assessed against existing codes to identify commonalities. Codes exhibiting similar patterns were subsequently grouped together into themes [47]. The research team reviewed codes and engaged in discussion to reach consensus on the themes.

Through the synthesis of included studies [27], 14 themes were inductively identified to address the first review aim of identifying barriers and facilitators. Whilst all could be mapped to at least one domain of the TDF, some were applicable to more than one domain. In these instances, a decision was made through discussion with the research team as to the most appropriate domain to allocate the finding. To improve clarity and to aid the development of specific recommendations, final results were organised and presented as barriers and facilitators in a tripartite structure of 1) Parental factors 2) Therapist factors 3) Organisational context and resources. These were then mapped to the BCW to address the second review aim of identifying relevant practice recommendations.

## Results

### Characteristics of studies

**Figure 3.**
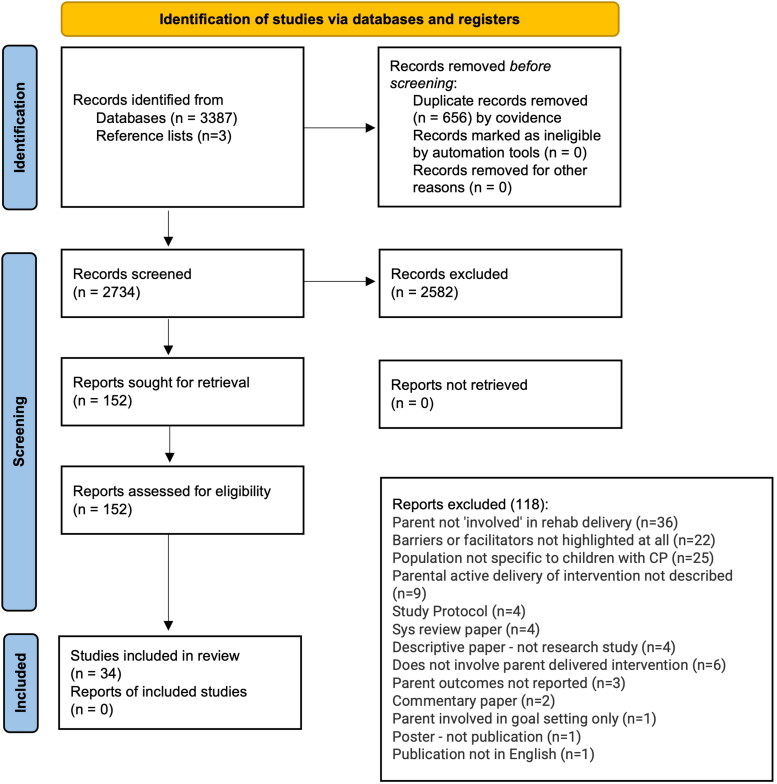
PRISMA flow diagram.

Initial searches resulted in a total of 3390 references (Figure 3). Of these, 656 were duplicates and 2734 were screened based on their title and abstract. The resulting 152 articles were screened against the stated inclusion criteria. In total, 34 empirical studies were included for analysis.

The characteristics of the included studies can be found in supplementary material (appendix 2). Research was conducted in the United States of America (USA) (*n* = 5), the Netherlands (*n* = 5), United Kingdom (UK) (*n* = 4), Australia (*n* = 4), Sweden (*n* = 3), Norway (*n* = 2), New Zealand (*n* = 2), and Canada (*n* = 2). Single papers were retrieved from Israel, Brazil, Portugal, Saudi Arabia, Spain, Turkey, and Taiwan. Fourteen papers were qualitative, 19 quantitative (including 12 randomised control trials, 4 cohort studies, and 3 surveys) and one non-randomised experimental study. Seven systematic reviews were identified, and their reference lists were checked which resulted in three additional studies.

Overall, the included study participants totalled 29 healthcare professionals and 765 parents. Twenty-six of the papers included studies whose population only comprised children diagnosed with CP. Seven included children who were at risk of CP. One study [32] featured children with CP alongside three participants who were diagnosed with either a heart defect, down syndrome or developmental delay. Interventions covered by the included papers were early interventions (*n* = 7), intensive upper limb interventions; bimanual and modified constraint induced movement therapy (*n* = 8), treadmill training (*n* = 1), goal-oriented training (*n* = 3), intensive lower limb interventions (*n* = 3) and other home programmes (*n* = 12), which often lacked specific detail on content. Most interventions were focused on motor skill acquisition. Details are shown in Appendix 2 – study characteristics.

### Critical appraisal

Common strengths of the included studies were the use of relevant data collection methods to address their stated aims. Limitations were found in 9 of 15 qualitative studies in terms of an inadequate consideration or reporting of ethical issues such as taking consent [32, 34, 36, 37, 48–52]. However, studies with potential ethical ‘insufficiencies’, for example, through unclear descriptions, can be included if their findings contribute significant insights to the topic of interest [53]. In such cases, any ethical inconsistencies were noted in the quality appraisal (Appendix 3). In 11 of the 12 randomised control trials (RCT’s), participants were not blinded to the interventions [11, 12, 54–62]. In all 12 RCTs, therapists were not blinded to the interventions [11, 12, 54–63], although outcome assessors were blinded in all trials. Only one RCT did not describe the randomisation process. Participant groups appeared to be well matched in all studies. A common weakness of cohort studies was a lack of consideration with regard to confounding factors. Whilst the three surveys [44, 45, 52] had clear research questions with a study appropriate method, none used standardised measures which raises concerns as to the validity and reliability of their data. While all studies had some weaknesses, the impact of these on their findings was considered ‘noncritical’ rather than ‘fatal’ [64]. Therefore, although rigor of each study was considered as part of the synthesis process, none were excluded from the review based on a poor-quality assessment.

### Synthesis of results

**Figure 4.**
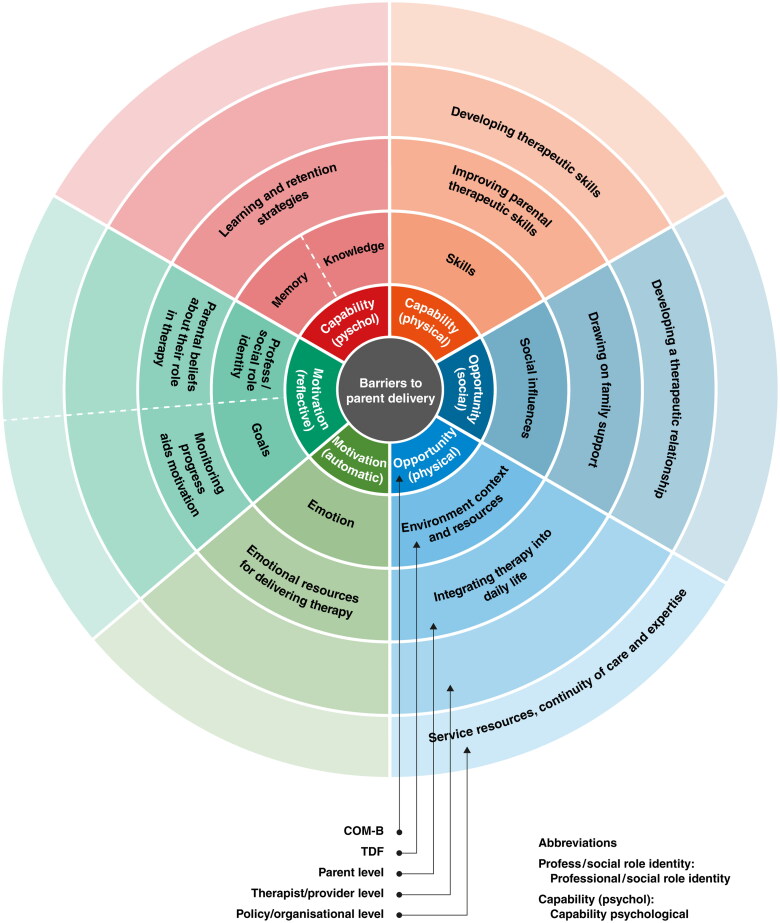
Barriers to parent-delivered interventions.

**Figure 5.**
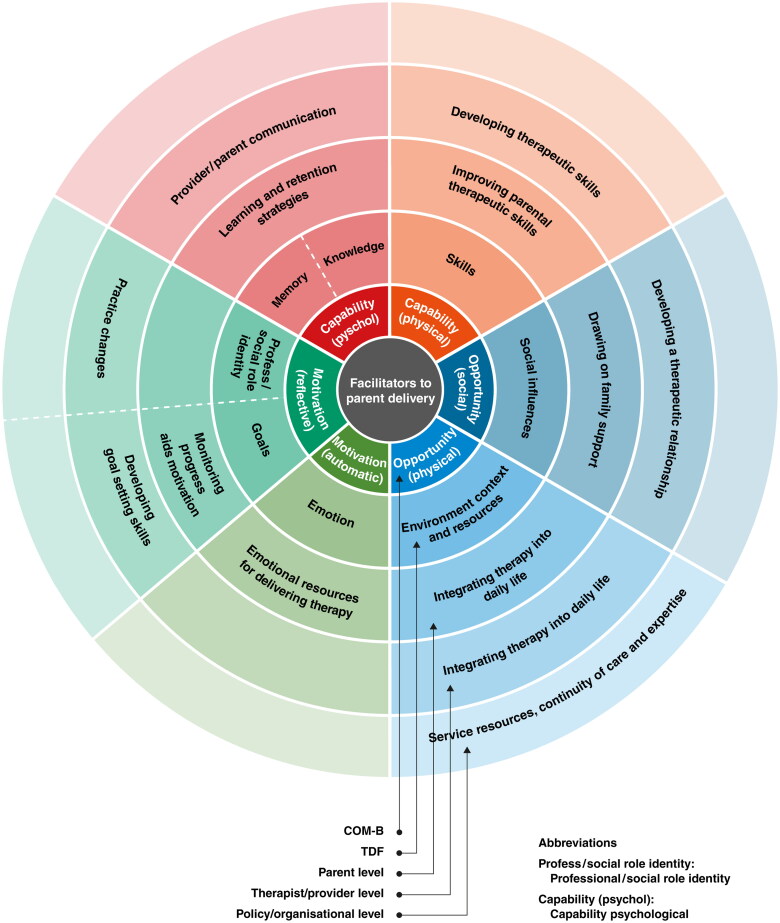
Facilitators to parent-delivered interventions.

Table 3 presents an integrated analysis with representative quotes highlighting the facilitators and barriers to capability, opportunity, and motivation from the perspectives of parents, therapists, and organisations. In the summary sections below, we highlight these in the context of the key themes identified in the review which are presented in italics. Figures 4 and 5 below summarise these findings.

**Table 3.** Integrated table of analysis.

COM-B/TDF	Theme	Facilitator source	Barrier source
**Parent factors**			
**CAPABILITY** (Psychological) TDF *Knowledge and memory*	Learning and retention strategies	Understand their child’s disability and knowing what they need to do, [37–40, 46–49]. Understanding of child’s capacity [50] Parents learn to adapt their behaviour moving from doing things for their child to *“let her do activities before we do them for her”* [56], p. 415 Being taught how to help [49] Written notes and action plans [36, 40, 47]. Coproduced resources can be tailored to parent learning preferences [37]. Carefully paced information including most salient points [52].	Need for increased knowledge of child’s abilities and disabilities [36–41, 46–48, 51, 52, 54] Too much detailed information was overwhelming and unhelpful [43].
**CAPABILITY** (Physical) TDF - *Skills*	Improving parental therapeutic skills	Observing therapists, asking questions and guided practice [56]. coaching for parents [42, 49, 51, 52, 57–60]. Higher levels of parent confidence [47, 12, 69]. Increased parent participation-built confidence in delivering therapy and a sense of parental empowerment [50, 51, 58, 61].	Lacking therapeutic skills [43, 47, 51]. Parents belief in their capability, preferring to *“leave it to the experts”* [42], p. 319. Observing a skilled therapist deliver the intervention and then replicate it [51].
**OPPORTUNITY** (Physical) TDF - Environmental context and resources	Integrating therapy into daily life	Individualised programmes devised in the home environment could become a “part of life” [47], p.203 and were easier to implement than a "list of activities” [47], p.204. provided by the therapist [43, 44, 47, 55, 58, 64–66]. Fixing a schedule [50] or selecting and adapting therapeutic suggestions to integrate them into home-life [43, 47, 55]. Involving siblings and calling the therapy programme “homework”, [47], p.203,50.	A common perception was that programmes were time consuming [40, 42, 43, 51, 57, 64] and that competing demands on parent’s time limited the physical opportunities to implement interventions at home’ [40, 43, 61] “incorporating the therapy into the planning and/or organization of my family” contributed to parent stress [57], p.83.
**OPPORTUNITY** (social) TDF Social influences	Drawing on family support	Positive support and involvement from family members [38, 39, 47, 51, 64, 68]. Support from family members and other parents can bolster parents’ motivation to persevere [38] and increase adherence to a programme [44]. Having people who know the child well and can help with delivery may also provide vital parental respite [36, 73].	Implementing home-based interventions may disrupt family dynamics [51, 71]. Mothers often carry the responsibility in some cultures [45] Fathers can have difficulty accepting their child’s disability [36].
**MOTIVATION** (Automatic) TDF Emotion	Emotional resources for delivering therapy	Negative emotions “Sense of guilt” [38], p.1121 or pressure during home visits when it was clear that they had not been involved, then motivated positive action [38, 39, 50]. Time and support to adjust to their child’s diagnosis [38, 51].	Feelings of shock, grief, anxiety, guilt, self-blame, and uncertainty about their child’s future [37–39]. Parents “felt anxious they were not able to do enough for their child” [51], p.564. Anxiety about *“doing something wrong at home”* [45], p.761. Stress can lead to burnout and poor adherence, as experienced by 21% of parents in an RCT of home delivered therapy [67]. Increased stress with intensive programme vs control group [69] Parent well-being needs to be considered particularly with families at high social risk and higher than average levels of anxiety and depression [47, 62].
**MOTIVATION** (Reflective) TDF – Professional/social role and identity	Parental beliefs about their role in therapy		Tensions between parents’ desires to meet their child’s therapeutic needs whilst retaining the parental role p [42, 43, 47, 50]. Cultural perceptions of intervention delivery being only the therapist’s role: “there are things therapists do - and things mothers do.” [43], p. 276.
TDF - *Goals*	Monitoring progress aids motivation	Monitoring progress through goal achievement [42]. Progress updates [47].	Reluctance to lead on goal setting, preferring to remain “only a parent” [42], p.318. Parents experience frustration, guilt, lack of confidence, irritation and may lose hope when unrealistic goals are set, therefore their child’s goals are not reached [38, 61, 64, 66, 68, 70].
COM-B	Theme	Facilitator source	Barrier source
**Therapist factors**			
**CAPABILITY** (Psychological) TDF - *Knowledge and Memory*	Provider-parent communication	Information delivered in manageable chunks [37–39] Giving a clear rationale for parental involvement increases their involvement [38, 43, 47, 12]. Listening and two-way communication [42].	
**CAPABILITY** (Physical) TDF - *Skills*	Developing therapeutic skills	Opportunities for parents to observe therapists delivering intervention and modelling interactions, guided practice, opportunities to ask questions and discuss the child’s development [36, 37, 43, 44, 47, 49, 62, 63]. Coaching to support parents in delivering programmes [49, 52, 59, 62, 63]	Therapists questioned their ability & lacked confidence to equip parents with the requisite therapeutic skills. [51].
**OPPORTUNITY** (Physical) TDF - Environmental context and resources	Integrating therapy into daily life.	Developing fun, flexible programmes tailored to the child, parents, and home environment [44, 47, 50, 58]. Individualised therapy approaches [37, 43, 44, 49, 50, 56, 58, 65–68].	
Social Opportunity TDF – *Social influences*	Developing a therapeutic relationship	Therapeutic relationships; when therapists “demonstrate a love for their child; an acceptable level of clinical expertise; and an emotionally supportive “friendship” [36], p. 83. Positive reinforcement and reassurance [39, 41, 43, 47, 49, 51] A strong trusting connection enabled honest communication [36, 38]. Talk about the child’s progress and advise on their child’s care [51]. An emotional investment in their child, not just focused on assessment [68]. Honest, transparent communication [42, 49].	Power dynamics within the therapeutic relationship “You appear to be wrongly assuming that the professional is in control and the parent is then encouraged to take part – when it is the parents’ life, child and future” [51], p. 564. Poor relationships may affect the parent’s comfort level in providing honest feedback [39].
**MOTIVATION** (Reflective) TDF - Professional/social role and identity	Practice changes	Shift behaviour to enhancing parental skill to implement their child’s intervention, [36, 59]. Acknowledge the parents’ contributions and be ready to listen to innovative and creative solutions [43]. Therapist role shift to educating and supporting the parent [52].	
TDF- *Goals*	Developing goal setting skills	Structured support in setting realistic achievable goals [42, 49, 52, 55, 62]. Therapist parent collaboration leads to more realistic goals. [39, 42, 43, 46, 47, 63] Regular reviews to provide feedback, adjust goals, [11, 55, 66, 68] and observe a child’s progress against goals set [51, 62, 65].	
**Organisational factors**			
**OPPORTUNITY** (Physical) TDF - Environmental context and resources	Service resources, continuity of care and expertise	Appropriate resources and programme flexibility [43, 47, 58, 64, 66] Adaptable service structure [38]. Time to develop good therapeutic relationships [39]. Staff retention and developing expertise [36, 47, 64]. Services with appropriately targeted support [39]. Fresh perspectives by changing therapists where positive relationships are not forged [36]	Scheduling home visits with working parents and children at school [66]. Regional variability, lack of services and lengthy waiting times [68]. Poorly resourced services and lack of staff [38, 64]. Staff turnover and changes of therapist [64]. Presence of too many professionals [38, 39].

#### Capability

Research under the theme of *Improving parental therapeutic skills* suggested that parents felt inadequately skilled in delivering intervention techniques, which would engage their child appropriately. However, there was also evidence that parent’s acknowledged their need to learn. They felt more confident and able to contribute to intervention delivery when they understood their child’s disability, and they were clear on what they could do as reflected in *Learning and retention strategies*.

Study findings under the theme of *provider-parent communication* suggested that therapists could support parents by providing programme material and intervention theory in a considered pace. *Developing therapeutic skills* theme comprised studies demonstrating that therapists’ use of coaching, modelling and guided practice alongside regular feedback could improve parent skills. However, studies suggested that therapists did not always feel confident about their ability to use techniques such as coaching.

#### Opportunity

Research under the theme of *Integrating therapy into daily life* highlighted the practical issues that made intervention delivery challenging for parents. Many reported a lack of time to practice and that the burden of delivery could interfere with family life. Several studies highlighted individualised programmes devised in the home were easier to integrate into their child’s routine rather than lists of activities. As reflected in the *Drawing on family support* theme, inclusion of the wider family often benefited parent-delivery in terms of increasing resources for practice, skills and motivation. However, implementing intervention may disrupt family dynamics.

Studies showed therapists were able to support *Integrating therapy into daily life*, by devising flexible, personalised and fun programmes which were easy to practice within the family context. Research under the theme of *Developing a therapeutic relationship* showed that therapists could foster parent-delivery by building a trusting, therapeutic relationship with the family, using honest communication and providing positive reinforcement.

However, therapists relationship building was often hampered by organisational barriers within the services in which they worked and were identified under the theme of *Service resources, continuity of care and expertise.* These included the impact of staff shortages which caused poor care continuity, lack of resources, inflexible appointment scheduling and inadequate time provided to bond with or teach families.

#### Motivation

Under the theme, *Emotional resources for delivering therapy,* research suggested that although some parents found delivery rewarding, others experienced negative emotions such as anxiety towards intervention delivery. Parents also acknowledged their discomfort about taking an active role and being a ‘therapist’ as well as a parent as shown in the theme, *Parental beliefs about their role in therapy*. Research from the theme *Monitoring progress aids motivation*, highlighted that whilst parents could be motivated by watching their children achieving programme goals, they were not encouraged when the goals were unrealistic or not perceived as meaningful. However, despite the clear benefits of being involved in setting these goals, many parents felt unprepared or unskilled to do this.

The importance of therapists evolving their practice to empower parents was demonstrated in the theme *Practice changes*. For example, shifting their focus from traditional intervention delivery to parental education, and coaching was key. Valuing parental contributions and acknowledging them as experts was central to creating an equitable partnership. Under the theme, *Developing goal setting skills,* studies highlighted therapists role in supporting collaborative goal setting to set realistic goals positively influences child outcomes, and that therapists need to prioritise collaborative goal setting by increasing parents’ confidence in this area.

## Discussion

The aim of this review was to identify the barriers and facilitators to parent-delivered interventions and to make recommendations for practice using established models of behaviour change. These factors were mapped to the COM-B model and TDF to develop greater understanding of how the behaviour of therapists and parents could be changed to improve outcomes. Recommendations were mapped to the BCW to inform practice.

The interventions in this review varied in relation to type and level of intensity and these factors may determine the extent to which they can be integrated into family life. For example, it may be easier to integrate individualised home programmes [65].rather than intensive approaches such as CIMT, mCIMT and bimanual therapy. The knowledge and skills required for both parents and therapists to deliver interventions also varied. In addition, their amount of relevant experience at delivering the interventions may impact on parental confidence and stress levels [57, 73]. Some articles also lacked adequate details of the content of the interventions and the parental education required [58,61].

The literature identified in this review was of mixed quality with a number of small lower quality studies [34, 52, 57, 62] but also several robust trials [12, 38, 12, 59, 65]. Some articles also lacked adequate details of the content of the rehabilitation interventions and parental education included [36, 60]. Given the greater number of parents contributing to the data set compared with therapists, findings were skewed towards parental needs for support and education. In addition, whilst using the COM-B was a useful way of framing behavioural components, some findings could have potentially fitted into several domains. This reflects the relationships between the central aspects of the model: capability, opportunity and motivation, and the complexity of parent-delivered interventions.

However, the use of the COM-B model highlighted the importance of enhancing psychological capability (i.e. knowledge), physical capability (i.e. skills) and motivation for both parents and therapists, of providing a conducive environment to increase the likelihood of parent-delivered interventions and gave further insight into the role of social opportunity (i.e. therapeutic relationships and family support). The behaviour change wheel may be used to improve parent-delivered interventions through identifying intervention functions to address the target barriers and facilitators. For example, to enhance aspects of psychological and physical capability, strategies such as education, training, restructuring the environment and modelling could be useful. Increasing social opportunity may be addressed through enablement strategies such as support from family and other parents in similar position. Furthermore, where reflective motivation is deemed of high importance, strategies such as persuasion, knowledge and incentivisation could be useful.

The review highlighted lack of parental confidence in intervention delivery which can be improved by providing them with information on their child’s condition and appropriate delivery skills. The review supports previous research in CP and other childhood conditions which suggests that parental coaching can effectively develop parent’s competence [76–80]. Research suggests that an increase in parent’s mastery boosts their sense of empowerment and can also improve their psychological health [56]. Individualised programmes delivered by parents in the home enable practice to be embedded into the child’s routine. Consideration should be given to competing demands on parent’s time and wider family dynamics [20, 62, 81].

The review suggests several ways that therapists can facilitate parent-delivered interventions. They can devise fun, individualised programmes in the home context, incorporate opportunities for parents to observe them delivering therapy and provide a rationale for what they are doing. Information needs to be provided in an appropriately paced way, using clear action plans, and co-produced or tailored using multi-media resources for effective learning [18, 35]. Previous research has suggested that therapist transparency increases parents’ trust and confidence, encourages realistic goal setting and leads to greater intervention practice [62, 82, 83]. Consistent with other research, this review also found that collaborative goal setting can enhance the therapeutic partnership, improve parental reflective motivation and increase competency [82–85]. Collaborative goal setting does not always occur in practice; and there is a need for therapists to develop their skills and capabilities in this area, while also educating parents on setting goals for their children [86, 87]. Interventions involving education, training, persuasion and enablement may support such practice change.

Therapists need to continue to move towards therapeutic approaches which facilitate family involvement and educate parents [19, 20]. They need to feel comfortable about this aspect of their role. Providing clearer clinical guidance and training for therapists on how to support parents will increase their confidence and the likelihood that such an approach will be incorporated into practice. Indeed, therapists would also benefit from formalised training in educational and motivational techniques to help them coach parents effectively [88].

Despite the importance of the therapeutic relationship between parent and therapist for parental delivery [89], it is created within the confines of healthcare services which do not always foster optimal relationships. Frequent changes in staffing hinder the development of rapport and lead to a lack of continuity. Therapists also need to be able to allocate sufficient appointment time both to build relationships and adequately role model intervention delivery for parents. Services should look to alternative ways of assisting parents, for example, through connecting them with families in similar situations to provide social support in a less resource intensive way [62].

Finally, parental-delivery of interventions should be encouraged, it must not be at the expense of over-burdening the family with unwanted responsibility [90]. Therapists need to ensure that they clearly understand parents’ expectations, motivations and capacity prior to implementing interventions [17, 91, 92]. Intervention programmes have to be developed whilst considering the wider context of the whole family [81] Parenting a child with CP has been associated with significant childcare burden and burnout over time [93–96] and the impact of advocating for and seeking services are contributory factors to this [81]. Parental needs or vulnerabilities must be considered when determining the extent of their involvement with delivery. Being offered the opportunity to deliver interventions may help parents feel that they are actively contributing to their child’s progress. Equally, participating in an intensive intervention programme can adversely affect parents and siblings [97–99]. Minimising any negative impact on family dynamics is a key consideration.

### Strengths and limitations

This is the first integrative review of both qualitative and quantitative studies to identify barriers and facilitators to parent involvement in delivering therapeutic interventions to children with or infants at high risk of CP. Through the further application of COM-B and TDF, the behaviour domains related to these identified barriers and facilitators have been highlighted to enhance practice relevance. This review applied clear inclusion criteria and employed a comprehensive search strategy. However, limitations should be considered when interpreting the findings. Firstly, only one reviewer performed the literature search. Although this may have had implications for its rigour, eligibility criteria were checked with co-authors prior to the search, whilst data extraction was undertaken by two authors. Secondly, the eligibility criterion of studies with >80% of children with or infants at high risk of CP was applied to ensure the validity of data for therapists and parents. However, this decision may have excluded some relevant perspectives on parent engagement in other clinical areas which might be useful for therapists working with CP populations. The literature identified in this review was dominated by mothers, with fathers’ perspectives rarely elicited. The review did not exclude articles of lower quality, as despite their flaws, they covered areas of inquiry not addressed by higher quality studies. Finally, only articles published in English were included in the review, meaning that potentially eligible non-English language studies may have been omitted.

### Implications for research and practice

Further research is required to advance our understanding of the impact that delivering interventions for children with or infants at high risk of CP has on parents. This research should focus on the impact of delivery on their wellbeing, feelings of competence and self-efficacy. Fathers’ voices have been rarely heard in studies to date and thus greater attention should be paid to their experiences of intervention delivery. Indeed, given the role that the wider family play in ensuring the success of parent-delivered interventions, it will be important to involve more relatives in future research. In addition, the cultural, economic and social diversity of participants were rarely reported in research. These factors should be considered when developing parent-delivered interventions to ensure they align with the values and preferences of different families. Intervention research would also be enhanced by using an agreed reporting framework to ensure the appropriate description of the rehabilitation programmes and parent training associated with them [100]. There is a clear need to develop evidence-based support for parents who choose to deliver their child’s intervention programme. When developing such supportive interventions, researchers may benefit from using an experience based co-design approach which involves stakeholders from the outset [101, 102]

Our analysis using behaviour change theory provides clear implications for advancing practice in this area. Services need to focus on enhancing the psychological capability of parents and therapists through the use of appropriate coaching and education programmes. Providing education for parents which is targeted appropriately will increase their confidence and competence at intervention delivery. By increasing the physical and social opportunities for collaboration and communication, therapists can create a productive working partnership with families which will foster progress and improve motivation for all. Finally, when considering parent-delivered interventions a holistic approach needs to be taken to address family needs and inimize parental burden [95,96].

## Conclusion

This review suggests that parents who are delivering interventions for their child with or infant at risk of CP will benefit from developing salient knowledge and delivery skills, which in turn, will grow parental confidence. Individualised, flexible programmes which are motivating for the child and tailored to their daily life, family and community context are fundamental to achieving positive child outcomes. Highly skilled therapists who understand parental needs, along with responsive and resourced healthcare services, are key to this process.

## Supplementary Material

Supplemental Material

## Data Availability

Data sharing is not applicable to this article, as no new data were created or analysed in this study.
