# Supplementary material for: Barriers and facilitators to parent-delivered interventions for children with or infants at risk of cerebral palsy. An integrative review informed by behaviour change theory
Source: Disabil Rehabil. 2024 Apr 16;47(2):287–301. doi: 10.1080/09638288.2024.2338193 (PMC11716669; doi:10.1080/09638288.2024.2338193)
Supplement: Supplemental Material [file IDRE_A_2338193_SM5693.zip › Revised_Supplementary Material_Appendix 2_Study characteristics.docx]

Supplementary Material

Appendix 2 - Study characteristics

| **Author / Year / Country**  **Objectives** | **Participants** | **Intervention type & location** | **Parent role in intervention delivery** | **HCP role supporting parent** | **Design** | **Data Collection & Measures** | **Data Analysis** | **Findings theme** |
| --- | --- | --- | --- | --- | --- | --- | --- | --- |
| Alwhaibi et al 2022 [52]  Saudi Arabia  To determine the factors that influence Saudi mothers’ compliance with HEP for their  C-CP | 113 mothers of children with CP | Home Exercise Program (physiotherapy)  No further details  Home based only | Delivery of prescribed home exercise programme –  length and time not specified | Not stated | Survey | Self-administered online questionnaire | Descriptive statistics  Chi-square for associate between adherence and demographic data. | Integrating therapy into daily life (p-f & t-f)  Drawing on family support (p-f)  Developing therapeutic skills (t-f) |
| BasAran et al 2014 [44]  Turkey  To determine the factors that affect the adherence to home exercise programs among caregivers of children with cerebral palsy | 147 Caregivers of children with CP (2=18years old)  Spastic Diplegia n=54  Spastic Hemiplegia n-39  Spastic Quadriplegia n-50  Other n-4  GMFCS  II=37  II = 21  III=32  IV=24  V=33 | Daily home exercise programme (no further details provided)  Home based only. | Delivery of prescribed home exercise programme –  length and time not specified | Unclear description  States: caregiver was instructed on a home exercise programme. | Survey | Adherence survey (researcher designed)  Beck Depression inventory (BDI)  Beck Anxiety inventory (BAI) | Descriptive statistics | Emotional resources for delivering therapy (p-b)  Integrating therapy into daily life (t-f) |
| Basu et al 2017 [34]  UK  To develop a manualised parent-delivered home-based early therapy intervention for the first 6 months of life to improve motor function in infants with predominantly unilateral perinatal stroke | 9 Parents of children with unilateral CP due to perinatal stroke  16 HCP’s (community and hospital paediatric physiotherapists and occupational therapists, neonatal physiotherapists, a play specialist and a paediatric neurodisability consultant. | Intervention  ‘eTIPS’ Early Therapy in Perinatal Stroke  Home based only | Apply principles to everyday activities | Parental support: monthly visits at home. Fortnightly texts from research team – reinforce messages, troubleshoot & positive feedback & encouragement | Qualitative | Focus groups | Normalisation Process Theory (NPT). | Learning and retention strategies (p-f; p-b)  Emotional resources for delivering therapy (p-f)  Provider- parent communication (t-f)  Developing therapeutic skills (t-f)  Integrating therapy into daily life (t-f) |
| Basu at al 2018 [35]  UK  Aim: To assess feasibility of the intervention and to pilot the outcome assessments prior to proceeding to randomised control trial (RCT) | **51 Parents / caregivers** (P/C; M=mothers; F=fathers; GM = grandmother; GF Grandfather)  **26 Infants**  6 perinatal stroke (P/C=6 M; 6 F; 1GM)  7 unilateral haemorrhagic parenchymal infarct (HPI); (P/C: 7M; 5F; 1GF)  13 typically developing.  (P/C: 13M; 12F)  **6 Therapists;** | Intervention  ‘eTIPS’ Early Therapy in Perinatal Stroke  Parent delivered home based early therapy intervention.  Co-produced instructional DVD and manual | Home  Apply principles to everyday activities | Parental support:  Home visits and text messages. | Feasibility trial | Feasibility questionnaire  In depth interviews  Paediatric Stroke Outcome Measure (PSOM)  Alberta Infant Motor Scale (AIMS)  Hand Assessment Infants (HAI)  General Movements (GM’s)  Accelerometry  ETIPS Feasibility  Parenting Sense of Competence Scale (PSOC)  Warwick Edinburgh Mental Well being scale (WEMWBS)  Questionnaire  Questionnaire for therapists | Descriptive statistics  Grounded theory | Drawing on family support (p-f)  Monitoring progress aids motivation (p-b)  Integrating therapy into daily life (t-f)  Developing a therapeutic relationship (t-f)  Developing goal setting skills (t-f)  Service resources continuity of care and expertise (o-b) |
| Beckers et al 2021 [71]  *(Schnackers et al 2018 protocol)*  Netherlands  To explore child and parent related effects of the home based bimanual training programmes in children with unilateral cerebral palsy | 14 children (2-7 years) median age 4 years with unilateral CP;  GMFCS I = 13  GMFCS II = 1 | Intervention  Goal oriented task specific training  Instructional video and manual | Home –  dosage 3.5 hours p/w for 12 weeks | Therapists coached parents in applying training.  (1 x face to face then mostly telephone; 3 x home visits) A remedial educationalist supported parents in relation to parent-child interaction and child behaviour. Coaching provided by professionals from child’s rehab centre. | Case Series | Canadian Occupational Performance Measure (COPM)  Hand Use at Home Questionnaire (HUH)  Lifestyle Assessment Questionnaire for CP (LAQ-CP)  Goal Attainment Scaling (GAS)  Assisting Hand Assessment (AHA)  Observational Skills Assessment Score (OSAS)  Parent Semi Structured Interviews | Descriptive Statistics  Thematic analysis | Improving parental therapeutic skills (p-f)  Integrating therapy into daily life (p-b) |
| Chamudot et al 2018 [53]  Israel  To examine the efficacy of mCIMT in treating infants younger than 18 months diagnosed with spastic hemiplegic CP by comparing it with a conventional non-constraining bimanual treatment of equal intensity | 33 Infants with hemiplegia (mean corrected age 11.1 months SD 2.2) | Intervention  Modified Constraint Induced Movement Therapy (mCIMT) (n=17)  /  Comparison  bimanual therapy (n=16) Both interventions included home programmes for daily bimanual play-based sessions: | Home only  Dosage – 1 hour per day for 8 weeks. | Therapist provided guidance 1 x p/w at home | Randomised controlled trial | mini Assisting Hand Assessment (mini AHA)  Functional Inventory  Parenting Sense of Competence Scale (PSCS)  Feasibility questionnaire | Inferential statistics | Improving parental therapeutic skills (p-f)  Integrating therapy into daily life (p-f); (t-f)  Service resources continuity of care and expertise (o-f) |
| Eliasson et al 2018 [11]  *(Eliasson et al 2014 study protocol)*  Sweden  To explore the effectiveness of baby-cimt (constraint-induced movement therapy) vs baby massage for improving manual ability of infants with unilateral cerebral palsy | 37 Infants 3-8months with asymmetric hand function and at risk of developing unilateral CP  At 1 year of age; 31 children diagnosed unilateral CP; 18 received baby CIMT& 13 received baby massage | Intervention  Baby Constraint Induced Movement Therapy (CIMT)  /  Comparison  Baby massage | Home intervention – assessments at hospital  Parents will be trained  CIMT  30min / day 6 days p/w for 12 weeks (total dosage 36 hours) given folder of materials.  Baby Massage  1 x p/d; 6 days p/w 12 weeks (72 occasions) | Monitoring through home visits (baby CIMT)  Coaching at weekly home visits. | Randomised parallel group trial | Hand Assessment for infants (HAI)  Assisting Hand Assessment (AHA)  Parent Sense of Competence Scale  Feasibility questionnaire | Descriptive statistics | Developing goal setting skills (t-f) |
| Ferre et al 2015 [75]  United States of America (USA)  To determine the feasibility of H-HABIT in terms of compliance, caregiver perception of difficulty in completing the activities and the impact of the intervention on the psychosocial dynamic between caregivers and their child (i.e. caregiver stress); | 11 Children (30-54 months)  Unilateral spastic CP | Intervention  Home Hand-Arm Bimanual Intensive Therapy  (H-HABIT) | Home based 90 hours  2 hrs p/d 5 days p/w 9 weeks  Caregiver training 3 sessions  Weekly 1 hour home visits during intervention | Helped to design individualised program for child | Single Group Design  (feasibility) | Feasibility  Parenting Stress Index Short form (PSI-SF)  AHA  Canadian Occupational Performance Measure (COPM) | Inferential statistics | Drawing on family support (p-b) |
| Ferre et al 2017 [54]  United States of America (USA)  To examine the efficacy of caregiver-directed, home-based intensive bimanual training in children with unilateral spastic cerebral palsy (USCP) using a randomized control trial | 24 Children/young people with unilateral cerebral palsy (2yrs 6mo – 10yrs 1mo) | Intervention  H-HABIT  /  Comparison  Lower Limb Intensive Functional Training (LIFT) | Home based  Caregiver directed activities 2 hrs p/d 5 days p/w for 9 weeks (total 90 hours) | Caregivers trained over 2 sessions. Supervision 1hr p/w for 9wks. | Randomised controlled trial | Box and Blocks Test (BBT)  AHA  COPM | Inferential statistics | Integrating therapy into daily life (p-f)  Drawing on family support (p-b) |
| Harrison et al 2007 [32]  Canada  Parent experiences and stories, determining which factors hinder, and which facilitate their ability to learn from their therapists. | 9 mothers  Diagnoses of children:  6 - CP  1 heart defect  1 down syndrome  1 developmental delay | Not specified – parents actively involved in implementing therapist recommendations | Community based therapy | Not specified | Qualitative | Semi structured face to face interviews | Thematic analysis | Learning and retention strategies (p-f) (p-b)  Developing therapeutic skills (t-f)  Developing a therapeutic relationship (t-f)  Practice changes (t-f)  Service resources, continuity of care and expertise (o-f) |
| Hielkema et al 2011 [33]  *(Hielkema 2010 protocol)*  Netherlands  To compare coaching based programme versus typical infant physiotherapy (traditional neurodevelopment techniques physical therapy) | 46 Infants (corrected age 3-6months)  High risk of developmental disorders defined by abnormal general movements  At 18mo diagnoses: 10 infants with spastic CP (2 unilateral and 8 bilateral)  29 infants complex minor neurological dysfunction;  5 minor neurological dysfunction  2 infants not reassessed and therefore no diagnosis given | Intervention  COPing and Caring for Infants with Special Needs (COPCA)  /  Comparison:  Traditional Infant Physiotherapy (TIP) (traditional neurodevelopment techniques physical therapy)  (Early intervention) | COPCA – at home  2 x p/w at home (mean no of sessions 33)  TIP – Frequency and location varied. Mean no of sessions 14 | Twice per week a home | Randomised controlled trial | Infant Motor Profile (IMP) | Descriptive statistics | Improving parental therapeutic skills (p-f); |
| Hielkema et al 2020 [56]  Netherlands  To compare family and functional outcomes in Coaching based EI programme versus typical infant physiotherapy (traditional neurodevelopment techniques physical therapy) | 43 Infants at very high risk of CP  Diagnosis at 21 months:  22 – CP  19 – No CP  2 – Unknown | Intervention  COPing and Caring for Infants with Special Needs (COPCA) n=23  /  Comparison:  Traditional Infant Physiotherapy (TIP) (traditional neurodevelopment techniques physical therapy) n=17  (Early intervention) | One year  COPCA at home frequency 3.0 (1.8-4.0)  TIP usually at home sometimes in outpatient setting frequency 2.5 (1.3-4.3) |  | Randomised controlled trial | IMP  Family Empowerment Scale (FES)  Nikmeegse Ouderliijke Stress Index questionnaire short version (NOSI-K)  Utrecjtse Coping List  Pediatric Evaluation of Disability Index (PEDI) Dutch version  Infant and Toddler Quality of Life Questionnaire (ITQOL) | Descriptive statistics | Improving parental therapeutic skills (p-f);  Developing therapeutic skills (t-f)  Practice changes (t-f) |
| Hinojosa & Anderson 1991 [51]  United States of America (USA)  To explore mothers’ experiences with and reactions to home treatment programmes | 8 mothers of preschool children with CP. | Home programmes –designed by therapist. | Not included | Not specified | Qualitative research | In depth interviews | Ethnographic analysis | Learning and retention strategies (p-f)  Improving parental therapeutic skills (p-b)  Integrating therapy into daily life (p-f; p-b; t-f)  Parental beliefts about their role in therapy (p-b)  Provider-parent communication (t-f)  Developing therapeutic skills (t-f)  Developing a therapeutic relationship (t-f)  Practice changes (t-f)  Developing goal setting skills (t-f)  Service resources, continuity of care and expertise (o-f) |
| Holmstrom et al 2019 [57]  Sweden  To evaluate small steps programme with standard care | 38 Infants at risk of CP or other neurodevelopmental disorders  Diagnosis at 2 years:  20 diagnosed CP  2 Autistic Spectrum Disorder  13 other neurodevelopmental disorders  2 no diagnosis development in or near typical range | Intervention  Small Step Programme  /  Comparison  standard care in infants | Home based.  Small step focus each lasting 6 wks; included 6 home visits hand use and mobility 4 home visits communication;  Parents providing daily basis  Parents coached and supervised by therapists;  Standard care provided by physiotherapist at hospital and parents advised on home training. | Coaching and supervision given to parents | Randomised controlled trial | The Peabody Developmental Motor Scales  GMFM-66  PEDI (self-care, mobility and social/cognitive)  Bayley Scales Infant Motor Development (cognition, language and motor) | Descriptive statistics | Learning and retention strategies (p-f; p-b)  Improving parental therapeutic skills (p-f)  Developing therapeutic skills (t-f)  Practice changes (t-f)  Developing goal setting skills (t-f) |
| Hurd et al 2022 [58]  Canada  Does early, intensive, child-initiated therapy for the lower extremity in children with perinatal stroke result in greater improvements in motor function than usual care? | 34 Children with confirmed perinatal ischaemic stroke and early signs of hemiparesis. | Intervention – intensive child initiated play based movement of lower extremities.  Laboratory delivered vs parent trained cohort | Parent trained cohort = intervention delivered by parent at home, visiting laboratory for assessments | Therapist guide parents; Weekly phone support and monthly in person collaborative training session | Randomised controlled trial | Gross Motor Function Measure-66; kinematics of walking; | Linear regression; descriptive statistics | Integrating therapy into daily life (p-f)  Developing goal setting skills (t-f) |
| Kruijsen-Terpstra et al 2016 [50]  Netherlands  To explore experiences and needs of parents and young children with CP regarding their child’s physical and occupational therapy process in a rehabilitation setting | 21 Parents of children (age 2-4 years) with CP | Physical and or occupational therapy in rehabilitation setting – 2 groups  Child focussed  Context focussed  Regular care | Rehabilitation setting |  | Qualitative research | Semi-structured interviews | Thematic analysis | Improving parental therapeutic skills (p-f; p-b)  Integrating therapy into daily life (p-b)  Drawing on family support (p-f)  Emotional resources for delivering therapy (p-b)  Parental beliefs about their role in therapy (p-b)  Monitoringprogress aids motivation (p-f; p-b)  Developing a therapeutic relationship (t-f)  Developing goal setting skills (t-f) |
| Lin et al 2011 [59]  Taiwan  Comparison of dose-matched home based control intervention with constraint - assess for differences in unilateral and bilateral motor performance, daily functions and quality of parental well being. | 21 Children with CP (age 48 – 119 months) | Home based constraint induced therapy / dose  matched home based control intervention (functional unilateral or bilateral arm training based on functional oriented activities, neurodevelopmental training and motor learning principle) | Home based    Children wear of elastic bandage on less affected hand & wrist for 3.5 to 4 hours p/d for 4 weeks. Parents encouraged to do exercises or daily activities | HCP’s delivered 3.5-4 hours per day 2 x p/w 4 weeks | Randomised controlled trial | PDMS-2  Bruininks-Oseretsky Test of Motor Proficiency (BOTMP)  Pediatric Motor Activity Log (PMAL)  Parenting Stress Index (PSI) Short form | Descriptive statistics | Emotional resources for delivering therapy (p-b) |
| Lowes et al 2014 [74]  United States of America (USA)  Does CIMT when compared to a usual care condition improve infants’ fine motor and gross motor performance?  Does CIMT when compared to a usual care condition improve parents’ report of infants’ functional performance when using the affected UE? | 5 Infants diagnosed with unilateral CP (7-18 months old) | CIMT  /  Usual care  Intervention  Home based.  CIMT  (non-affected arm cast 24 hrs p/d 23 days with 4 days no cast at end)  2 hrs p/d over 4 wks with occupational therapist (20hrs)  1 hr p/d over 4 weeks of  Comparison  Standard care 1 hour p/w therapy in outpatient clinic 4 weeks (functional tasks, play, sensory strength building and bilateral activities to promote use of affected arm/hand) | Parent implemented home programme  Parent education to promote use of affected upper extremity | Therapist provide 2 hrs p/d  Parent provide 1hr p/d | Pilot study (pre-test – post-test cohort design) | Bayley Scales of Infant Toddler Development-3 (BSID)  Infant motor activity log (IMAL)  Fidelity measure | Descriptive statistics | Improving parental therapeutic skills (p-f) Integrating therapy into daily life (t-f) |
| Mattern-Baxter et al 2013 [62]  United States of America (USA)  To compare intensive home treadmill training with standard care | 12 Infants; Children/young people with CP  (5 Hypotonic  7 Spastic Hemiplegic or diplegic) | Treadmill training (home)  /  regular home-based physiotherapy | Home Based  Intervention  Treadmill  10-20mins 2 x p/d; 6 x p/w for 6 wks carried out by parents  Weekly supervision from physiotherapist  Comparison  Weekly physiotherapy session at home | Weekly supervision from physical therapist | Non-randomised experimental study | Gross Motor Function Measure-66 (GMFM-66)  Pediatric Evaluation of Disability Inventory (PEDI)  Timed 10 min walk test (10MWT)  Functional Mobility Scale (FMS) | Descriptive statistics | Integrating therapy into daily life (p-f)  Monitoring progress aids motivation (p-b)  Integrating therapy into daily life (t-f)  Developing goal setting skills (t-f)  Service resources, continuity of care and expertise (o-f) |
| Morgan et al 2015 [60]  *(Morgan et al 2014 protocol for 2015,2016)*  Australia  To compare game programme intervention to standard care | 13 infants 3-5 months of age with abnormal general movements assessment score between 11-18 weeks post term age. | Goals Activity Motor Enrichment (GAME)  /  Standard care  (Early intervention) | Home based  GAME  Goal oriented intensive motor training.  Home visits min fortnightly with home programme. Involves; active motor learning, family centered care, parent coaching and environmental enrichment.  Standard care  Variable (average = up to 14 hours in first year of life) | Home visits at least fortnightly;  Parents coached in strategies to enhance child development. and trained in simple motor analysis; taught to optimise opportunities for learning | Randomised Controlled trial | The Peabody Developmental Motor Scales (PDMS-2)  Canadian Occupational Performance Measure (COPM)  Bayley Scales of Infant and Toddler Development (BSID-III)  Gross Motor Function Measure-66 (GMFM-66) | Descriptive statistics | Provider-parent communication (p-f) |
| Morgan et al 2016 [63]  Australia  To compare game programme intervention to standard care | 30 infants  corrected age 3-4 months with absent fidgety movements on general movements assessment or 5-6 months with diagnosis of CP | Goals Activity Motor Enrichment (GAME)  /  Standard Care  (Early intervention) | Home based  GAME  Goal oriented intensive motor training.  Home visits min fortnightly with home programme. Involves; active motor learning, family centered care, parent coaching and environmental enrichment.  Comparison  Standard care: Varied approaches combining clinic based sessions and home programmes some also receiving home visits. | Home visits at least fortnightly  Parents coached in strategies to enhance child development. and trained in simple motor analysis; taught to optimise opportunities for learning | Randomised Controlled trial | The Peabody Developmental Motor Scales (PDMS-2)  Canadian Occupational Performance Measure (COPM)  Bayley Scales of Infant and Toddler Development (BSID-III)  Gross Motor Function Measure-66 (GMFM-66) | Descriptive statistics | Emotional resources for delivering therapy (p-b)  Developing therapeutic skills (t-f)  Developing goal setting skills (t-f) |
| Morgan et al (2023) [66]  Australia  Aim: To investigate the views of parents of infants who received an early diagnosis of CP by immediate intensive GAME intervention | 10 parents of Infants aged 3-6 months corrected age, diagnosed with CP or “high-risk of CP” identified through general movements assessment | Goals Activity Motor Enrichment (GAME)  (Early intervention) | Home based intensive motor training, coaching and education of parents, assistance to modify the environment to promote learning. | Home visits at least fortnightly  Parents coached in strategies to enhance child development. And trained in simple motor analysis; taught to optimise opportunities for learning | Qualitative research | Interviews |  | Learning and retention strategies (p-f)  Improving parental therapeutic skills (p-f)  Developing therapeutic skills (t-f)  Integrating therapy into daily life (t-f)  Developing a therapeutic relationship (t-f)  Developing goal setting skills (t-f) |
| Myrhaug & Ostensjo 2014 [45]  Norway  To describe motor training and physical activity among  pre-schoolers with cerebral palsy (CP) in Norway, and assess associations between child,  parent, and motor intervention characteristics, and parent-reported child benefits from  interventions. | 360 parents | Goal directed intensive training incorporated into daily routines |  |  | Survey | Survey | Descriptive statistics | Learning and retention strategies (p-f; p-b)  Developing goal setting skills (t-f) |
| Novak 2011 [65]  Australia  To describe the experiences and views of parents who participated in our randomized controlled trial on partnership home programs | 10 Parents of children with CP  (5 x spastic diplegia; 1 spastic hemiplegia; 1 ataxia; 1 athetosis) mean age 7.7 years | Partnership home programmes | Home programmes | Establish collaborative partnership | Qualitative research | Semi structured interviews | Grounded theory | Learning and retention strategies (p-f; p-b)  Improving parental therapeutic skills (p-b)  Integrating therapy into daily life (p-f)  Drawing on family support (p-f)  Emotional resources for delivering therapy (p-b)  Parental beliefs about their role in therapy (p-b)  Monitoring progress aids motivation (p-f)  Provider-parent communication (t-f)  Developing therapeutic skills (t-f)  Integrating therapy into daily life (t-f)  Developing a therapeutic relationship (t-f)  Developing goal setting skills (t-f)  Service resources, continuity of care and expertise (o-f) |
| Odman et al 2007  [49]  Sweden  To evaluate parents’ perceptions of the service quality of the two training programmes, Lemo and Move&Walk, and to explore the association between defined factors included in a previously published framework for patients’ evaluation process and parents’ perceptions of the service quality. | 48 Parents of children with CP (spastic dyskinetic and ataxic CP) | Intensive training programmes (LEMO and Move & Walk) | 4 week intensive training programme  LEMO 3 hours p/d 4 days p/w (average 14 days)  Move and Walk  2-4 hours p/d for 4to 5 hours p/w (average 15 days) |  | Qualitative research | Telephone interviews (question responses based on a 4-point likert scale)  Patient perspective on care and rehabilitation process (POCR) | Descriptive statistics | Learning and retention strategies (p-b)  Developing a therapeutic relationship (t-f) |
| Palomo_Carrion et al 2022  [69]  Spain  To determine the feasibility of the home-application of 80 h of BIT for 8 weeks (2 h/day: 5 days per week: 80 h) in children with spastic unilateral cerebral palsy (SUCP), aged 5–8 years-old with low and very low performance, being conducted by parents. | 10 children with spastic unilateral CP | Bimanual training and modified Constraint Induced Movement Therapy | 2 days (4 hrs p/d) of family training provided – intervention explained and activities provided  Home only  Dosage 80 hrs bimanual followed by 40 hrs CIMT – total 100 hrs over 10wks. | Weekly online follow up  Prior to intervention family training provided 8 hours (over 2 days) & included: intervention explanation & example with activities. Child preferences were sought from parents.  Therapists created list aligned to child preferences as example for parents | Feasibility | Single Group Design | Anova Test and Bonferroni correction. | Learning and retention strategies (p-b) |
| Peplow & Carpenter 2013  [72]  UK  To explore how parents of children with cerebral palsy, attending mainstream primary schools, perceive the relevance  of, and adherence with, exercise programs | 4 (one father and 3 mothers) | Prescribed exercise programme | Carried out by parents and school staff |  | Qualitative research | Semi structured interviews (face to face) | Inductive thematic analysis | Integrating therapy into daily life (p-f; p-b)  Drawing on family support (p-f)  Emotional resources for delivering therapy (p-b)  Service resources continuity of care and expertise (o-f; o-b) |
| Pereira Domenech 2016  [48]  Portugal  Mothers perspective of the role of physiotherapy in the treatment of children with CP, AND mothers' compliance with physiotherapists' recommendations at home. | 11 mothers of children with CP | Home based physiotherapy programmes | Home based (no further details provided) | Not specified | Qualitative research | Semi structured interviews | Content analysis | Learning and retention strategies (p-f; p-b)  Integrating therapy into daily life (p-b)  Drawing on family support (p-b) |
| Piggot et al 2002  [36]  New Zealand  To identify describe and generate a conceptual model of the experience of parents and therapists taking part in home therapy programmes for children with cerebral palsy. | 8 Parents of children with CP (2 hemiplegia 5 quadriplegia)  4 Therapists (3 x physiotherapists & 1 x occupational therapist) | Home therapy programmes  (individually designed encompass range of activities including; stretching, positioning in standing frames, eye tracking toys and assisted cruising) | Home therapy programme  no dosage & variety of activities | Not specified | Qualitative research | Semi structured interviews | Grounded theory | Learning and retention strategies (p-f; p-b)  Drawing on family support (p-f; p-b)  Emotional resources for delivering therapy (p-f; p-b)  Provider-parent communication (t-f)  Developing a therapeutic relationship (t-f)  Service resources, continuity of care and expertise (o-f; o-b) |
| Piggot et al 2003  [37]  New Zealand  Therapists’ and parents’ perspectives of the key issues, concerns and their experiences with regard to home programs | 8 Parents of children with CP (2 hemiplegia 5 quadriplegia)  4 Therapists (3 x physiotherapists & 1 x occupational therapist) | Home therapy programmes  (Individually designed; encompass range of activities including; stretching, positioning in standing frames, eye tracking toys and assisted cruising) | Home therapy programme  no dosage & variety of activities | Not specified | Qualitative research | Semi structured interviews | Grounded theory | Learning and retention strategies (p-f; p-b)  Drawing on family support (p-f)  Emotional resources for delivering therapy (p-f; p-b)  Provider-parent communication (t-f)  Developing a therapeutic relationship (t-f; t-b)  Developing goal setting skills (t-f)]  Service resources, continuity of care and expertise (o-f; o-b) |
| Ross and Thomson 1993  [68]  UK  To evaluate parents’ involvement in the management approach as carried out by a community paediatric physiotherapist in the two inner London health authorities. | Parents of 38 preschool children with CP | Home physiotherapy programme | Mostly home based programme implemented by parents |  | Survey | Questionnaires | Descriptive statistics | Learning and retention strategies (p-b)  Improving parental therapeutic skills (p-f; p-b)  Integrating therapy into daily life (p-b)  Drawing on family support (p-f)  Emotional resources for delivering therapy (p-f; p-b)  Developing therapeutic skills (t-b)  Developing a therapeutic relationship (t-f; t-b)  Developing goal setting skills (t-f) |
| Saquetto 2018  [61]  Brazil  to assess whether the addition of an educational programme for primary caregivers to rehabilitation improves functioning in children with cerebral palsy. | 63 children with cerebral palsy (1-12 years) | Intervention  Conventional rehabilitation (NDT) plus primary caregiver education programme  /  Comparison  standard rehabilitation (NDT) delivered by a rehabilitation practitioner | Conventional rehabilitation = 1 x p/w 30 mins for 12 wks  Caregiver education 1 x p/w 45mins for 12 weeks group | Therapist design individualised programme.  Intensive coaching by a multidisciplinary team with regard to therapeutic content and implementation of training in daily life. | Randomised controlled trial | Gross motor function measure Pediatric Evaluation of Disability Inventory (PEDI) | Descriptive statistics | Learning and retention strategies (p-f; p-b) |
| Smidt 2020  [38]  Norway  To explore parents’ and occupational therapists’ experiences with a home program using goal-directed training to improve hand function in daily activities for children with bilateral cerebral palsy | 5 Parents of children with bilateral CP;  3 Occupational Therapists | Goal Directed Training | Home based  Implemented by parents 40 hours over 8 weeks | Establish collaborative partnership | Qualitative research  Focus Groups | Intervention outcomes  Canadian Occupational Performance Measure (COPM)  Goal Attainment Scaling (GAS) | Content analysis | Learning and retention strategies (p-f)  Integrating therapy into daily life (p-f)  Developing goal setting skills (t-f)  Service resources, continuity of care and expertise (o-b) |
| Verhaegh 2021  [67]  Netherlands  To evaluate parents’ experiences with the home-based training program using a video coaching approach in order to optimize implementation strategies | 13 parents of children with unilateral CP | Constraint induced movement therapy / bimanual therapy  Combined:  2 x appointments at rehabilitation centre. Parent home delivered intervention video coaching (8 wks) | 8 week blocks of intensive home based upper limb training  Dose (28hrs) 30mins p/d  7 days p/w  8 weeks | Remote video coaching. Coaching consisted of written feedback and suggestions  Parents trained at rehab centre in how to conduct training at home.  Resources provided: instruction booklet (photos and seating instruction) Box of toys matching child’s hand function and developmental stage of play. Toys demonstrated to parent  Video coaching – written feedback on videos provided by parent. | Qualitative | Interviews | Inductive thematic content analysis | Learning and retention strategies (p-f)  Integrating therapy into daily life (p-f; t-f)  Emotional resources for delivering therapy (p-f)  Parental beliefs about their role in therapy (p-b) |
