# Supplementary material for: Barriers and facilitators to parent-delivered interventions for children with or infants at risk of cerebral palsy. An integrative review informed by behaviour change theory
Source: Disabil Rehabil. 2024 Apr 16;47(2):287–301. doi: 10.1080/09638288.2024.2338193 (PMC11716669; doi:10.1080/09638288.2024.2338193)
Supplement: Supplemental Material [file IDRE_A_2338193_SM5693.zip › Revised_Supplementary Material_Appendix 3_Quality Appraisal.docx]

**Key to the following tables:**  ✓ = yes X = no ? = Can’t tell

Critical Appraisal Skills Programme - Qualitative Studies [30].

|  | **1. Was there a clear statement of the aims of the research?** | **2. Is a qualitative methodology appropriate?** | **3. Was the research design appropriate to address the aims of the research?** | **4. Was the recruitment strategy appropriate to the aims of the research?** | **5. Was the data collected in a way that addressed the research issue?** | **6. Has the relationship between researcher and participants been adequately considered?** | **7. Have ethical issues been taken into consideration?** | **8. Was the data analysis sufficiently rigorous?** | **9. Is there a clear statement of findings?** | **10. How valuable is the research?** |
| --- | --- | --- | --- | --- | --- | --- | --- | --- | --- | --- |
| Alwahaibi  2022  Saudi Arabia  [52] | ✓ | ✓ | ✓ | ✓ | ✓ | ? | ✓ | ✓ | ✓ | Determining factors Compliance with home programmes |
| Basu et al  2017  UK  [34] | ✓ | ✓ | ✓ | ✓ | ✓ | ? | ✓ | ✓ | ✓ | Valuable –intervention development for children from 6 months |
| Harrison et al (2007)  Canada [32] | ✓ | ✓ | ✓ | ✓ | ✓ | X | ? | ✓ | ✓ | Focus only on mothers perspectives. |
| Hinojosa  1991  USA  [51] | ✓ | ✓ | ✓ | ? | ✓ | X | ? | ✓ | ✓ | Very useful insights into parent perspectives |
| Kruijsen Terpstra  2016  Netherlands  [50] | ✓ | ✓ | ✓ | ✓ | ✓ | ? | ✓ | ✓ | ✓ | one specific rehab setting, explores parent and includes child experience |
| Morgan 2023  Australia  [66] | ✓ | ✓ | ✓ | ✓ | ✓ | ✓ | ✓ | ✓ | ✓ | Valuable parent insights into experiences of early intervention |
| Novak  2011  Australia [65] | ✓ | ✓ | ✓ | ✓ | ✓ | ✓ | ✓ | ✓ | ✓ | Highly relevant |
| Odman  2007  Sweden [49] | ✓ | ✓ | ✓ | ? | ✓ | X | ✓ | ? | ✓ | Evaluating parent perceptions. |
| Peplow and Carpenter  2013  UK [72] | ✓ | ✓ | ✓ | ✓ | ✓ | ✓ | ✓ | ✓ | ✓ | Adds to knowledge of parent perceptions |
| Pereira Domenech et al  2016  Portugal [48] | ✓ | ✓ | ✓ | ✓ | ✓ | ? | ✓ | ? | ✓ |  |
| Piggot et al  2002  New Zealand [36] | ✓ | ✓ | ✓ | ✓ | ✓ | ? | ✓ | ✓ | ✓ | Valuable parent insights into experiences |
| Piggot et al  2003  New Zealand [37] | ✓ | ✓ | ✓ | ✓ | ✓ | ? | ✓ | ✓ | ✓ | Valuable parent insights into experiences |
| Smidt  2020  Norway [38] | ✓ | ✓ | ✓ | ✓ | ? | ✓ | ✓ | ✓ | ? |  |
| Verhaugh  2021  Netherlands  [67] | ✓ | ✓ | ✓ | ✓ | ✓ | ✓ | ✓ | ✓ | ✓ | Adds to knowledge of parent experience home based training |

Critical Appraisal Skills Programme - Randomised Control Trials [32]

|  | 1. Did the RCT study address a clearly focused research question | 2. Was the assignment of participants to intervention randomised? | 3. Were all participants who entered the study accounted for at its conclusion? | 4a. Were the participants “blind” to intervention they were given? | 4b. Were the investigators “blind” to the intervention they were giving to participants? | 4c. Were the people assessing/ analysing outcome(s) “Blinded”? | 5. Were the study groups similar at the start of the randomised controlled trial? | 6. Apart from the experimental intervention, did each study group receive the same level of care? | 7. Were the effects of intervention reported comprehensively? | 8. Was the precision of the estimate of the intervention or treatment effect reported? | 9. Do the benefits of the experimental intervention outweigh the harms and costs? | 10. Can the result be applied to your local population/in your context? | 11. Would the experimental intervention provide greater value to the people in your care than any of the existing interventions? |
| --- | --- | --- | --- | --- | --- | --- | --- | --- | --- | --- | --- | --- | --- |
| Chamudot et al  2018  Israel [53] | ✓ | ✓ | ✓ | X | X | ✓ | X | ✓ | ? | ✓ | ? | ? | X |
| Eliasson  2018  Sweden [11] | ✓ | ✓ | ✓ | X | X | ✓ | ✓ | X | ✓ | ? | ✓ | ✓ | ✓ |
| Ferre  2017  USA [54] | ✓ | ✓ | ✓ | X | X | ✓ | ✓ | ✓ | ✓ | ✓ | ? | ✓ | ✓ |
| Hielkema  2011 Netherlands [33] | ✓ | ✓ | ✓ | ? | X | ✓ | ✓ | ? | ✓ | X | ? | X | ? |
| Hielkema  2020  Netherlands [56] | ✓ | ✓ | ✓ | X | X | ✓ | ✓ | ✓ | ✓ | ✓ | ? | ✓ | X |
| Holmstrom  2019  Sweden [57] | ✓ | ✓ | ✓ | X | X | ? | ✓ | ✓ | ✓ | ✓ | ? | ✓ | ? |
| Hurd  2022  Canada [58] | ✓ | ✓ | ✓ | X | X | ✓ | X | X | ✓ | ✓ | ? | ✓ | ? |
| Lin  2011  Taiwan [59] | ✓ | ? | ✓ | X | ? | ✓ | ✓ | ✓ | ✓ | X | ? | ? | ? |
| Mattern-Baster (2013) USA [62] | ✓ | ✓ | ✓ | X | X | ✓ | ✓ | ✓ | ✓ | ✓ | ? | ✓ | ✓ |
| Morgan  2015  Australia [60] | ✓ | ✓ | ✓ | X | X | ✓ | ✓ | X | ✓ | ✓ | ✓ | ✓ | ✓ |
| Morgan  2016  Australia [63] | ✓ | ✓ | ✓ | ✓ | X | ? | ✓ | ✓ | ✓ | ✓ | ✓ | ✓ | ✓ |
| Saquetto  2018  Brazil [61] | ✓ | ✓ | ✓ | X | X | ✓ | ✓ | ✓ | ✓ | ✓ | ? | X | ? |

Critical Appraisal Skills Programme - Cohort studies [31]

|  | Did the study address a clearly focused issue? | Was the cohort recruited in an acceptable way? | Was the exposure accurately measured to minimise bias? | Was the outcome accurately measured to minimise bias? | Have the authors identified all important confounding factors? | Have they taken account of the confounding factors in the design and/or analysis? | Was the follow up of subjects complete enough? | Was the follow up of subjects long enough? | What are the results of this study? | How precise are the results? | Do you believe the results? |
| --- | --- | --- | --- | --- | --- | --- | --- | --- | --- | --- | --- |
| Beckers et al (2021) Netherlands  [71] | ✓ | ✓ | ✓ | X | ? | X | ✓ | ✓ | Bimanual home programmes with coaching for children with unilateral CP | Causality not able to be assumed; small sample and limitations in outcome measures | To be interpreted with caution, but findings align with other reports. |
| Ferre (2015)  USA  [75] | ✓ | ✓ | ? | ✓ | X | X | ✓ | ✓ | Home based bimanual therapy is feasible | Small sample unable to draw definitive conclusions | While a small sample size findings align with other reports |
| Palomo-Carrion (2022) Spain  [69] | ✓ | ✓ | X | ✓ | ? | ? | ✓ | X | Home based family delivery of BIT and mCIMT is feasible | Small sample | Limitations in sample size and |
| Lowes et al (2014)  USA  [74] | ✓ | ✓ | ✓ | ? | ? | X | ✓ | ✓ | The intervention was feasible to be delivered in an intensive home based model with positive effects for infants with unilateral CP | Use of non standardised measures; small sample and lack of blinding limit results | Limitations in results of this study |

ROBINS-I Tool [33]

|  | Bias due to confounding | Bias in selection of participants | Bias in classification of interventions | Bias due to deviations from intended interventions | Bias due to missing data | Bias in measurement of outcomes | Bias in selection of reported result | Overall risk of bias (low/moderate/  serious/critical) |
| --- | --- | --- | --- | --- | --- | --- | --- | --- |
| Basu (2018)  UK [35] | Moderate risk of bias | Low risk of bias | Moderate risk of bias | Low risk of bias | Moderate risk of bias | Low risk of bias | Low risk of bias | Low |

Critical Appraisal of a Survey Centre for Evidence-Based Management [34]

|  | Did the study address a clearly focused question / issue? | Is the research method (study design) appropriate for answering the research question? | Is the method of selection of the subjects (employees, teams, divisions, organizations) clearly described? | Could the way the sample was obtained introduce (selection) bias? | Was the sample of subjects representative with regard to the population to which the findings will be referred? | Was the sample size based on pre-study considerations of statistical power? | Was a satisfactory response rate achieved? | Are the measurements (questionnaires) likely to be valid and reliable? | Was the statistical significance assessed? | Are confidence intervals given for the main results? | Could there be confounding factors that haven’t been accounted for? |
| --- | --- | --- | --- | --- | --- | --- | --- | --- | --- | --- | --- |
| BaSaran  (2014) Turkey [44] | ✓ | X | X | ? | ✓ | ? | ✓ | X | ✓ | ✓ | ? |
| Myrhaug & Ostensjo (2014)  Norway [45] | ✓ | ✓ | ✓ | ? | ✓ | X | ✓ (34%) | X | ✓ | X | ? |
| Ross & Thomson (1993) UK [68] | ✓ | ✓ | ✓ | ✓ | ✓ |  | ✓ (60.5%) | X | ✓ | X | ✓ |
