# Supplementary material for: Barriers and facilitators to parent-delivered interventions for children with or infants at risk of cerebral palsy. An integrative review informed by behaviour change theory
Source: Disabil Rehabil. 2024 Apr 16;47(2):287–301. doi: 10.1080/09638288.2024.2338193 (PMC11716669; doi:10.1080/09638288.2024.2338193)
Supplement: Supplemental Material [file IDRE_A_2338193_SM5693.zip › Supplementary Material_Appendix 1_Medline Search Criteria.docx]

|  | cerebral palsy. |
| --- | --- |
|  | Brain injur* |
|  | ((bilateral or bi-lateral) adj3 spastic). |
|  | (quadripleg* adj3 spastic). |
|  | (diplegi* adj3 spastic). |
|  | (unilateral adj3 spastic). |
|  | (hemiplegi* adj3 spastic). |
|  | CP |
|  | 1 or 2 or 3 or 4 or 5 or 6 or 7 or 8 |
|  | (motor disorder* or neurodevelopmental disorder* or neurodisabilit* or motor skills disorders). |
|  | adolescen*. |
|  | infant*. |
|  | child*. |
|  | Disabled child* |
|  | (p?diatric* or neonat*) |
|  | 9 or 10 |
|  | 11 or 12 or 13 or 14 or 15 |
|  | (parent adj2 involve*). |
|  | (parent adj3 experience) |
|  | (parent adj3 engagement). |
|  | Family cent* |
|  | parent* or caregiver* or carer*). |
|  | 18 or 19 or 20 or 21 or 22 |
|  | Therap* |
|  | Intervention* |
|  | (Rehabilitation or habilitation) |
|  | Physi* therap* |
|  | Occupational therap* |
|  | 24 or 25 or 26 or 27 or 28 |
|  | (barrier* or facilitator)* |
|  | 9 and 16 and 17 |
|  | 23 and 31 |
|  | 29 and 31 |
|  | 30 and 33 |
|  | 31 or 32 or 33 or 34 |
